# Optimal Design of a Planar Textile Antenna for Industrial Scientific Medical (ISM) 2.4 GHz Wireless Body Area Networks (WBAN) with the CRO-SL Algorithm

**DOI:** 10.3390/s18071982

**Published:** 2018-06-21

**Authors:** Rocío Sánchez-Montero, Carlos Camacho-Gómez, Pablo-Luís López-Espí, Sancho Salcedo-Sanz

**Affiliations:** Department of Signal Theory and Communications, Escuela Politecnica Superior, Universidad de Alcala, Campus Universitario, Ctra. de Madrid a Barcelona km 33.600, 28805 Alcala de Henares, Spain; carlos.camacho@uah.es (C.C.-G.); pablo.lopez@uah.es (P.-L.L.-E.); sancho.salcedo@uah.es (S.S.-S.)

**Keywords:** electro-textile, wearable textile antenna, Industrial Scientific Medical (ISM), wireless off-body sensors systems, co-evolution Coral Reefs Optimization (CRO)

## Abstract

This paper proposes a low-profile textile-modified meander line Inverted-F Antenna (IFA) with variable width and spacing meanders, for Industrial Scientific Medical (ISM) 2.4-GHz Wireless Body Area Networks (WBAN), optimized with a novel metaheuristic algorithm. Specifically, a metaheuristic known as Coral Reefs Optimization with Substrate Layer (CRO-SL) is used to obtain an optimal antenna for sensor systems, which allows covering properly and resiliently the 2.4–2.45-GHz industrial scientific medical bandwidth. Flexible pad foam has been used to make the designed prototype with a 1.1-mm thickness. We have used a version of the algorithm that is able to combine different searching operators within a single population of solutions. This approach is ideal to deal with hard optimization problems, such as the design of the proposed meander line IFA. During the optimization phase with the CRO-SL, the proposed antenna has been simulated using CST Microwave Studio software, linked to the CRO-SL by means of MATLAB implementation and Visual Basic Applications (VBA) code. We fully describe the antenna design process, the adaptation of the CRO-SL approach to this problem and several practical aspects of the optimization and details on the algorithm’s performance. To validate the simulation results, we have constructed and measured two prototypes of the antenna, designed with the proposed algorithm. Several practical aspects such as sensitivity during the antenna manufacturing or the agreement between the simulated and constructed antenna are also detailed in the paper.

## 1. Introduction

Following a study of the U.S. Bureau of the Census [1], the number of elderly people (65–84 years old) will have doubled from 35 million to 70 million by 2025 in the USA alone. Of course, the healthcare expenditure will increase. The imminent health crisis is encouraging many researchers, industrialists and economist to focus on finding new optimal and quick health solutions based on technology. Since 2010 [2], IEEE 802 created a Task Group called IEEE 802.15.6 for defining the standardization of the new technology called Wireless Body Area Networks (WBAN). WBAN is an important technology advance because it supports a wide range of medical and Consumer Electronics (CE) applications, to provide real-time health monitoring of a patient without any restriction of ordinary life [3,4], environment survey and sport activities. According to the IEEE 802.15.6 standard, which was approved on 29 February 2012 [5], the elements that conform to the WBAN sensors should satisfy some specific constraints such as: low power consumption, low profile, compactness, integration on fabrics and the non-significant effect of the body on the characteristics. In WBAN, intelligent, miniaturized, low-power sensor nodes may be located in, on or around a human body to monitor its functions [6] and the surrounding environment [7]. In [5], several frequency bands have been defined for WBAN devices, such as the Medical Implant Communications System band (MICS: 400 MHz), the Industrial Scientific Medical Band (ISM: 2.4 GHz and 5.8 GHz) and the Ultra WideBand (UWB: 3–10 GHz). Recent advances in textile materials have allowed developing wearable electronics and antennas that are easy to integrate into garments [8,9]. In the literature, it is possible to find many textile antenna designs. Based on the wearable antennas application, they can be classified into two groups: on-body or off-body communications [10,11]. In the on-body communications, the data transmission takes place along the body between body-worn user nodes. In contrast, in the off-body communications, the data are transmitted from the body-worn device to an off-body device.

One of the most suitable antennas in off-body wireless body area communications is the Inverted-F Antenna (IFA) [12,13,14]. An inverted-F antenna is a planar metallic line printed on a PCB (Printed Circuit Board) with the ground plane etched in the same plane. The main advantage of this kind of antenna is their easy implementation and matching [13]. Moreover, the facts of etching the ground plane and having the radiator in the same side allow reducing radiation exposure to the human body, which is very important in WBAN services with textile materials. On the contrary, in terms of size, the IFA occupies quite a large space since its length is close to a quarter wavelength. Its miniaturization leads to a degradation of antenna bandwidth and efficiency [15,16]. However, the dimensions can be reduced adequately by folding [17], capacitive loading [18] or, as proposed in [12], using meander lines. Meander line technology allows designing antennas with a small size and provides good wideband performance [19]. Additionally, the use of meander line antennas was introduced to reduce the resonant length without great deterioration of its performance [20,21]. Usually, the width of the meander line and the spacing between meanders are set to a constant (unique) value. This simplifies the initial calculation and the further optimization process using classical methods. Note that when the meander line width and spacing are not unique, the number of design variables becomes very high, and then, classical optimization methods are no longer suitable. In that case, the employment of metaheuristic techniques has been shown to be very useful for antenna design [22,23,24].

In this paper, we propose a variation of a meander line IFA with a variable width and spacing meanders, optimized with a novel metaheuristic optimization algorithm for ISM applications. Another important part of our proposal is that the proposed antenna has been constructed considering a wearable substrate, which makes the designing process more difficult. In addition to the wearable substrate, the proposed antenna allows a good bandwidth and radiation pattern in WBAN working frequencies, which leads to a high performance in these systems. We propose to use a novel metaheuristic, the Coral Reefs Optimization with Substrate Layer (CRO-SL) [25], which has shown better performance than alternative metaheuristics such as Genetic Algorithms (GA) or Harmony Search (HS). The CRO [26,27] is an evolutionary-type algorithm, which simulates all the processes occurring in a real coral reef in order to carry out the optimization of a given system (the meander line IFA textile antenna in this case). The CRO-SL version has been successfully applied to a number of optimization problems previously [28,29], and it is able to combine different search patterns or strategies within a single population of potential solutions. In this case, we will show how this optimization scheme is able to obtain excellent results in the optimization process of the proposed antenna, tuning it for use in WBAN systems. In the experimental section of the paper, we also detail the accuracy of the antenna design (with the CRO-SL and simulation software), its construction and how it performs in terms of different measured parameters.

The rest of the paper is structured in the following way: the next section presents in detail the proposed antenna design, characteristics and variables to be optimized. Section 3 describes the proposed metaheuristics used to optimize the textile antenna. Section 4 shows the experiments carried out to optimize the antenna and the results obtained in the simulation and construction of the device. Finally, Section 5 closes the paper with some final remarks on the research carried out in this paper.

## 2. Proposed Antenna Design

The use of an IFA allows keeping the antenna and the ground in the same plane. Moreover, as previously mentioned, the meander shape has been used to reduce the final antenna dimensions [19]. Thus, an IFA with a Meander Shape (MS-IFA) seems to have the proper requirement to be used as n antenna for ISM applications. In order to have a starting point in the design of the MS-IFA, we have considered a modified version of an existing Printed Circuit Board (PCB) antenna prototype described in [30]. Note that this starting point device is not adjusted to 2.42 GHz, and in addition, the substrate considered to implement it is not textile material, so important changes of its design must be carried out in order to adapt it to use it in a wireless sensor for ISM systems. Unfortunately, there are no specific equations for the design of MS-IFAs, which makes it very difficult to tune the parameters to the optimal required values. This latter point leads to the use of metaheuristics approaches, as will be further described in the next sections.

**Figure 1:**
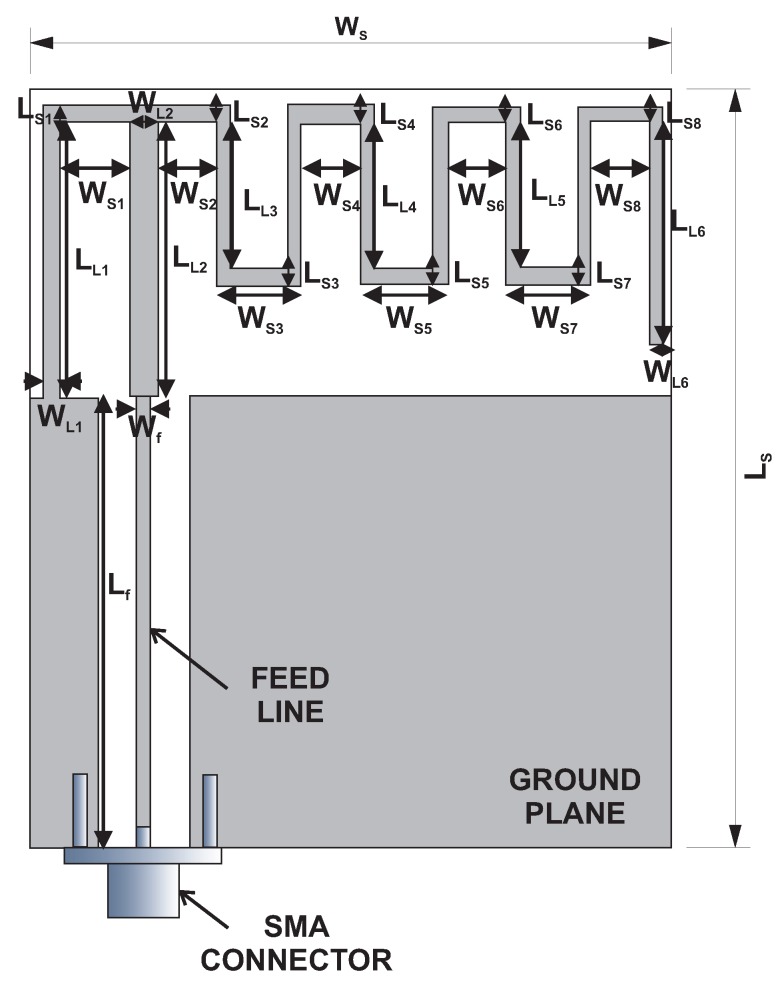
Geometry of the Meander Shape (MS)-Inverted-F Antenna (IFA) considered and the design variables.

In order to start the design phase, the first step consists of a simulation of the modified MS-IFA. For this, textile materials such as flexible foam ($\epsilon_{r}=1.3$) as the dielectric and flexible copper as the conductor, with thicknesses of 1.1 and 0.035 mm, respectively, have been considered. These materials allow an easy antenna implementation into wireless sensors for ISM, and therefore, its embedding in clothes such as life jackets, t-shirts, etc. [31,32]. The layout and design variables of the original antenna are shown in Figure 1, and the specific values of the initial antenna design (prior to optimization) are indicated in Table 1.

**Figure 2:**
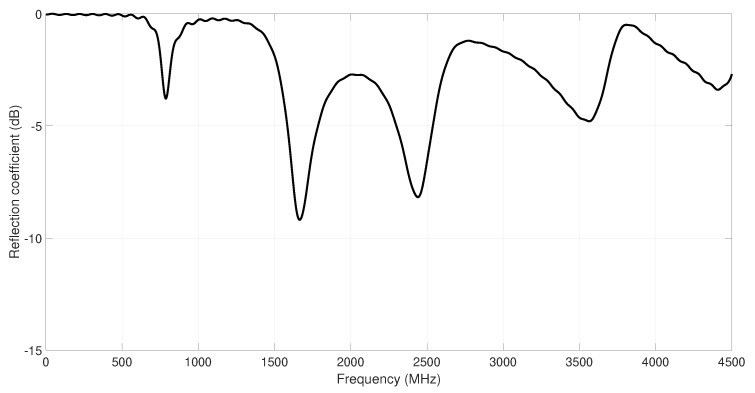
Reflection coefficient of the original antenna design over textile substrate (prior to optimization).

In this design phase, the antennas simulations have been carried out with CST Microwave Studio [33]. Figure 2 shows the reflection coefficient ($S_{11}$) obtained during the simulation process of the original MS-IFA (prior to optimization). According to these results, it is necessary to modify the dimensions of the antenna to achieve the resonant frequency at 2.42 GHz (to work in the ISM bandwidth). Moreover, it is also important to take into account the bandwidth requirement of wireless sensors for ISM services. According to [5], the reflection coefficient of the antenna sensors must be lower than −10 dB from 2.4–2.45 GHz. Thus, it is necessary to optimize the considered IFA meander line antenna to adjust its resonant frequency at 2.42 GHz and to get a bandwidth higher than 83 MHz. In order to fulfil these parameters of the design of the antenna, the objective function of the optimization process must be carefully selected, as shown in the next section.

### 2.1. Objective Function

The objective function is a key point of any optimization problem, since the algorithm uses it consistently in order to evaluate the quality of the solutions found. In metaheuristics and constructive algorithms, the objective function leads the search with continuous evaluations of different tentative solutions to the problem. In this case, the considered function (f(x)) to guide the antenna optimization must take into account different design requirements of the device, such as its resonant frequency and bandwidth, as was described in the previous section. Specifically, in order to calculate f(x), we first take into account a discretization of the $S_{11}$ antenna parameter, which will be calculated by the CST software. In this case, a discretization in steps of 2 MHz is considered. A measurement window between 2400 MHz and 2500 MHz is also selected to calculate f(x). The mathematical formulation of the objective function is the following:

(1) $$f\left(x\right)=0.8\times N^{-10\mathrm{db}}+0.1\times M+0.1\times M^{*}$$

where $N^{-10\mathrm{db}}$ stands for the number of $S_{11}$ points in the observation window under −10 dB, $M\;=\;|mean(S_{11})|$ and $M^{*}=\left|min\left(S_{11}\right)\right|$.

Note that this function takes into account the antenna bandwidth (within the first part of the equation) and also the resonant frequency and the actual value of the reflection coefficient by means of the second and third parts of the equation.

## 3. Methods: The Proposed Coral Reefs Optimization with Substrate Layer

This section presents the CRO-SL metaheuristic proposed in this paper for tackling the considered IFA meander line antenna design. First, the basic CRO algorithm is presented, which will be modified with a substrate layer in order to obtain a competitive co-evolution algorithm with different exploration procedures involved.

### 3.1. Basic CRO

The CRO [26,28] is an evolutionary-type algorithm, which simulates the processes occurring in a coral reef. The CRO algorithm first initializes some random positions of a simulated reef with random corals (solutions to a given optimization problem) and leaves some other positions empty. These holes in the reef are available to accommodate new corals at later stages of the algorithm. The rate between free/occupied positions in the reef at the beginning of the algorithm is a parameter of the CRO, denoted as $\rho_{0}$. In a second phase of the algorithm, the CRO simulates the processes of reproduction and reef formation.

1. External sexual reproduction or broadcast spawning: The simulation of the broadcast spawning mechanism of a reef is carried out as follows:
  - First, we select a random fraction of the existing corals, and they are assigned to be broadcast spawners. The fraction of broadcast spawners with respect to the overall amount of existing corals in the reef will be denoted as $F_{b}$.
  - Several coral larvae are then formed by applying a given crossover procedure between two broadcast spawners. Note that other exploration strategies can be applied.
2. Internal sexual reproduction or brooding: Brooding is another coral reproduction mechanism that is simulated in the algorithm. This reproduction is modeled by means of any kind of mutation technique and takes place on a fraction of corals of $1-F_{b}$. A percentage $P_{i}$ of the coral is mutated in every step of the algorithm.
3. Larvae setting: The larvae formed by the exploration mechanisms described above will try to set and grow in the reef. In this process, each larva randomly tries to settle in a position (i,j) of the reef, and if the location is free, it will achieve it. If the location is already occupied, the new larva will settle only if its fitness function is better than that of the existing coral. There is a maximum number of tries for a larva to settle down in the reef, η, after which the larva will be discarded.
4. Asexual reproduction: The CRO can also model asexual reproduction, in the following way: The whole set of corals in the reef are sorted according to their fitness value (given in this case by the objective function described in Section 2). Then, a small fraction (denoted as $F_{a}$) of the available corals is duplicated and mutated (with probability $P_{a}$) to provide variability and to try to settle down again in a different part of the reef as in Step 3.
5. Depredation: At the end of each reproduction iteration, a small number of corals in the reef can be depredated, thus liberating space in the reef for the next coral generation (iteration h+1). The depredation operator is applied with a very small probability ($P_{d}$) to a fraction ($F_{d}$) of the corals in the reef with worse health.

### 3.2. CRO with Substrate Layers

A new version of the CRO algorithm can be obtained by considering alternative processes that occur in a real coral reef. For example, different recent studies have shown that successful recruitment in coral reefs strongly depends on the type of substrate on which they fall after the reproduction process [34]. This specific characteristic of coral reefs was first included in the CRO in [35]. This new version of the CRO was named CRO-SL (Coral Reefs Optimization algorithm with Substrate Layers). In [25], a new version of the CRO-SL was presented, where each substrate layer provides a different search procedure for the algorithm. Following this version of the CRO, the general CRO-SL approach for competitive co-evolution was obtained, where each substrate layer represents different processes (different models, operators, parameters, constraints, repairing functions, etc.). The use of CRO-SL as a competitive co-evolution algorithm has been successfully tested in different applications such as micro-grid design [29], vibration cancellation in buildings [28,36] or in the evaluation of novel non-linear search procedures [37]. In this section, the main ideas of the CRO-LS as a co-evolution search algorithm are described, which will be applied to solve the antenna design problem tackled in this paper.

**Figure 3:**
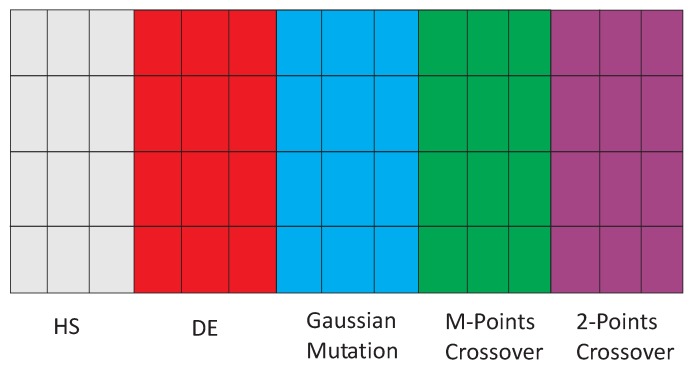
Example of different substrate layers in the Coral Reefs Optimization algorithm with Substrate Layers (CRO-SL) algorithm. HS, Harmony Search; DE, Differential Evolution.

The inclusion of substrate layers in the CRO can be done in a straightforward way, by dividing the reef into different zones. Each zone represents a different searching mechanism to be applied to the corals in that zone. This idea can be better understood with the example of Figure 3, which shows the new structure of the CRO-SL in an example with five different substrate layers. In this example, each substrate is assigned to a different exploration process, Harmony Search (HS), Differential Evolution (DE), Gaussian mutation, one-point crossover and two-point crossover.

### 3.3. Substrates Considered in the CRO-SL

The considered substrates in this paper to evolve the proposed modified MS-IFA are detailed below. Note that there are general purpose substrates, such as differential evolution or harmony search-based and other very specific substrates for real-encoding problems and with crossovers adapted to the problem at hand.

**Figure 4:**
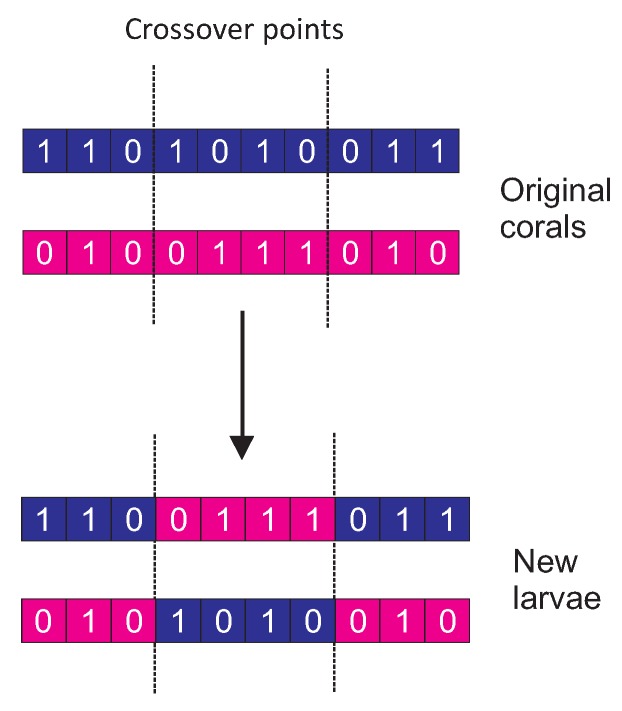
An illustration of the CRO broadcast scheduling process used to generate new larvae using the two-point crossover (2PX) substrate.

**Figure 5:**
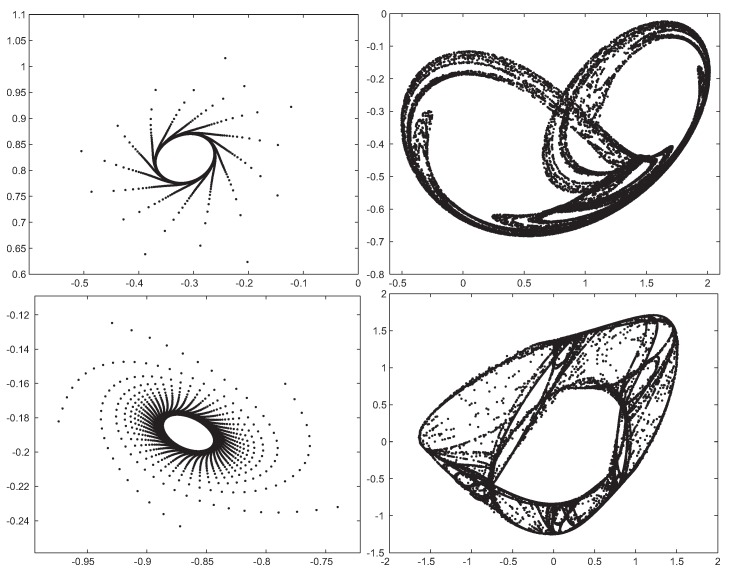
Examples of four strange attractors in the phase space (x vs. y), used in the SA substrate. Each pattern corresponds to a different set of parameters ($a_{1}-a_{12}$).

- Differential Evolution-based operator (DE): This operator is based on the evolutionary algorithm described in [38]. The DE introduces a differential mechanism for exploring the search space, in such a way that new larvae are generated by perturbing the population members using vector differences of individuals. Perturbations are introduced by applying the rule $v_{i}=x_{i}^{1}+F\left(x_{i}^{2}-x_{i}^{3}\right)$ for each encoded parameter on a random basis, where *v* corresponds to the output larva, $x^{t}$ are the considered parents (chosen uniformly among the population) and *F* determines the evolution factor weighting the perturbation amplitude.
- Harmony Search-based operator (HS): Harmony search [39] is a population-based Metaheuristic (MH) that mimics the improvisation of a music orchestra while it is composing a melody. HS controls how new larvae are generated in one of the following ways: (i) with a probability HMCR ∈[0,1] (Harmony Memory Considering Rate), the value of a component of the new larva is drawn uniformly from the same values of the component in the other corals; (ii) with a probability PAR ∈[0,1] (Pitch Adjusting Rate), subtle adjustments are applied to the values of the current larva, replaced with any of its neighboring values.
- Two-point crossover (2PX): 2PX [40] is considered one of the standard recombination operators in evolutionary algorithms. In the standard version of the operator, two parents from the reef population are provided as input. A recombination operation to from two larvae is carried out by randomly choosing two crossover points, interchanging then each part of the corals between those points, as schematically shown in Figure 4.
- Gaussian Mutation (GM): This operator is a variant of the classical random mutation method introducing a scaled Gaussian distribution [41]. Specifically, the Gaussian probability density function is:
  $$f_{G(0,\sigma^{2})}\left(x\right)=\frac{1}{\sigma \sqrt{2\pi }}e^{-\frac{x^{2}}{2\sigma^{2}}}.$$
  This operator has a mutation strength value $\sigma_{i}$ for every parameter of a solution, related to the lower and upper bounds $a_{i}$ and $b_{i}$, respectively. The mutated larva is thus calculated as: $x_{i}^{\prime }=x_{i}+\sigma N_{i}\left(0,1\right)$, where $N_{i}\left(0,1\right)$ is a random number following the Gaussian distribution.
- Strange Attractors (SA): SA is a search operator recently proposed in [37] and specifically designed to improve the searching capabilities of MHs by replicating different processes in nature known as fractal geometric patterns, as shown in Figure 5. In particular, it is designed to generate structures of non-linear dynamical systems with chaotic behavior [42]. These kinds of fractal structures can be generated by means of the general two-dimensional quadratic map:
  $$\left\{\begin{array}{c} x_{n+1}=a_{1}+a_{2}x_{n}+a_{3}x_{n}^{2}+a_{4}x_{n}y_{n}+a_{5}y_{n}+a_{6}y_{n}^{2}\\ y_{n+1}=a_{7}+a_{8}x_{n}+a_{9}x_{n}^{2}+a_{10}x_{n}y_{n}+a_{11}y_{n}+a_{12}y_{n}^{2} \end{array}\right.$$
  In order to introduce a chaotic mutation in a solution using SA, we followed the procedure introduced in [37]. Hence, a determined number of attractors S=50 is defined. Each time the operator is applied, a random attractor is considered. The quadratic map is calculated over a random number ϵ∈[500,1000] of iterations starting from an initial condition $\left(x_{0},y_{0}\right)=\left(0.6,0.9\right)$ until *x* and *y* are found. Then, the mutated larvae are obtained as:
  $$\left\{\begin{array}{cc} v_{i}=x_{i}+Fx; & p=1/2\\ or\\ v_{i}=x_{i}+Fy; & p=1/2 \end{array}\right.$$

## 4. Experiments and Results

**Figure 6:**
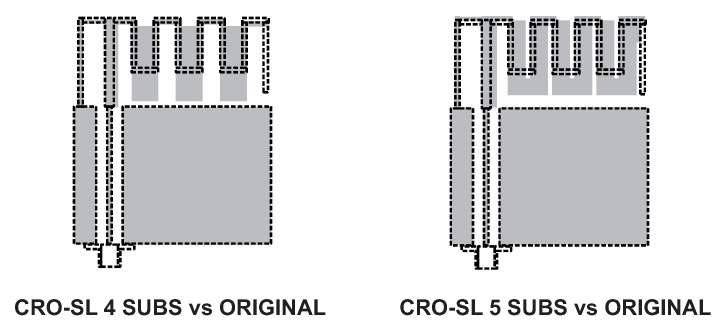
Original (dashed-line) and optimized MS-IFA (grey surface).

This section presents the experimental results obtained in the design of the proposed antenna with the CRO-SL approach. The CRO-SL algorithm’s main parameters used in all the simulations are shown in Table 2. Different experiments including different numbers of substrates in the CRO-SL algorithm were then performed in order to show the capabilities of the algorithm. The best results have been obtained by using the CRO-SL algorithm with four (all described in Section 3.3, but the HS) and five substrates (CRO-4SL and CRO-5SL, respectively). The final values for the MS-IFA after the optimization problem in both cases are shown in Table 3. The comparison between the original antenna (dashed-line) and the optimized MS-IFA (grey surface) is given in Figure 6.

**Figure 7:**
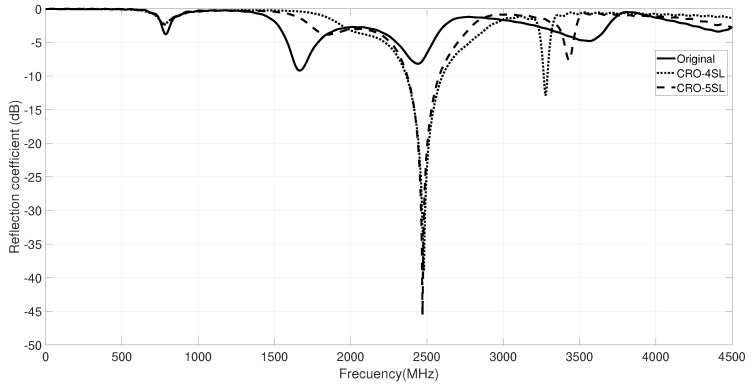
Reflection coefficients after the optimization process with the CRO-4SL and CRO-5SL.

The new responses for the reflection coefficient after the antenna optimization process with the CRO-4SL and CRO-5SL are represented in Figure 7. Note that the CRO-SL (both versions with four and five substrates) provide solutions with the resonant frequency of the antenna adjusted at 2.42 GHz, as defined in the required design. Moreover, the antenna bandwidth satisfies the off-body ISM service requirements in both cases.

**Figure 8:**
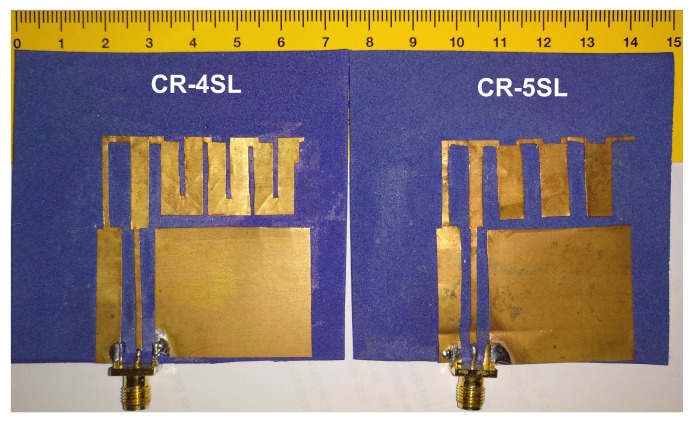
Final prototypes of the modified MS-IFA over the textile substrate constructed. On the left, the one obtained with the CRO-4SL. On the right, the one obtained with the CRO-5SL.

In order to validate the simulation results, both prototypes obtained with the CRO-4SL and CRO-5SL algorithms have been manufactured. They are shown in Figure 8.

**Figure 9:**
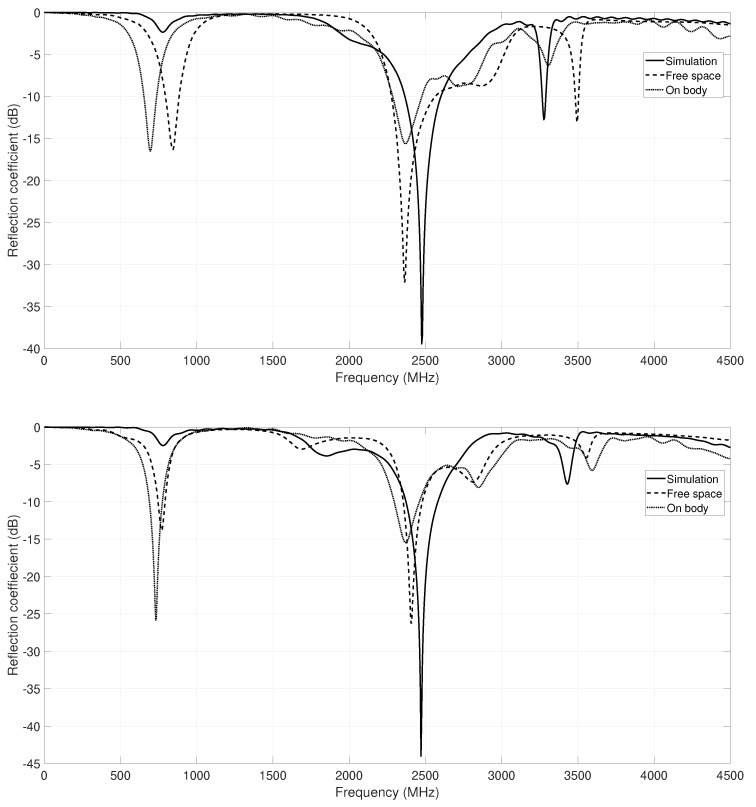
Reflection coefficient simulated and measured for prototypes obtained with the CRO-4SL and CRO-5SL: (**Top**) CRO-4SL prototype; (**Down**) CRO-5SL prototype.

Measurements of the reflection coefficient for both prototypes have been carried out, and they have been compared with the simulation results; see Figure 9a,b. As the figures show, the behavior of the antenna at the resonant frequency is very good, and in both cases, the bandwidth requirements are satisfied. However, note that the prototype antenna obtained with the CRO-5SL is better than its counterpart obtained with the CRO-4SL, since the former exhibits a wider bandwidth, and the values of the reflection coefficient are lower than for the antenna optimized with the CRO-4SL algorithm.

**Figure 10:**
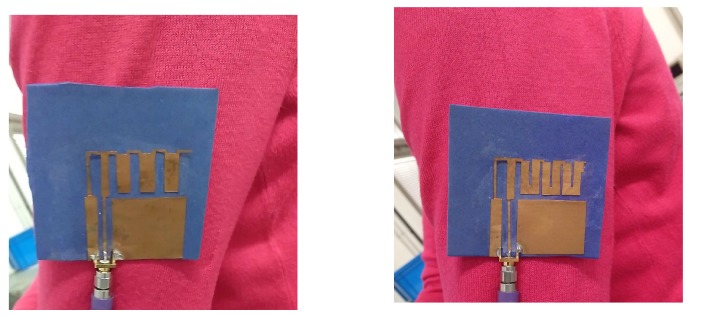
Measurement of the final prototypes on the body. (**Left**) the one obtained with the CRO-4SL; (**Right**) the one obtained with the CRO-5SL.

Moreover, to validate the effect of the body in these prototypes, a measurement of the reflection coefficient has been done as can be seen in Figure 10. The behavior of each antenna is shown in Figure 9. According to the results, the performance of the prototypes is appropriate to use in ISM applications, because the body interaction does not modify the radiation properties.

**Figure 11:**
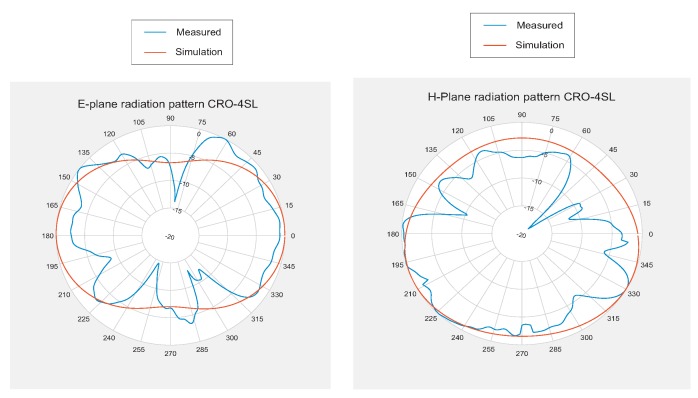
E-plane and H-plane H radiation patterns for the MS-IFA optimized with the CRO-4SL algorithm: (**Left**) E-plane; (**Right**) H-plane.

**Figure 12:**
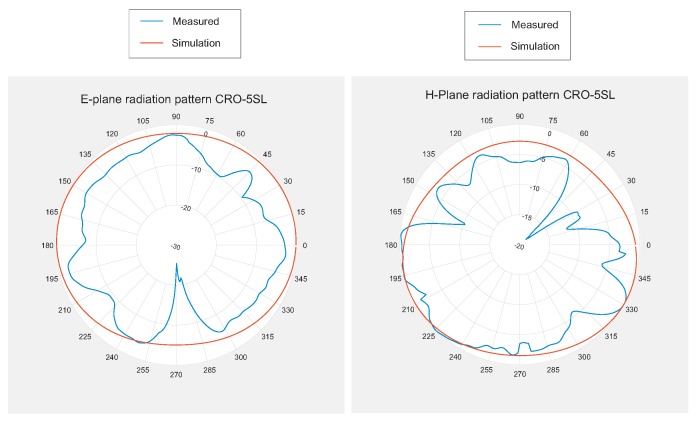
E-plane and H-plane radiation patterns for the antenna optimized with the CRO-5SL algorithm: (**Left**) E-plane; (**Right**) H-plane.

In addition to the reflection coefficient results, the radiation patterns of the constructed antennas have been analyzed and compared to those obtained by simulation. Figure 11 and Figure 12 show these results. In this case, the measurements were taken in an anechoic chamber, and we could not perform on-body measurements.

Regarding the simulation of the antennas’ gain values, in both cases, they are very similar, around 3.14 dBi. Note that when both antennas were measured, these values resulted in being lower than in the simulations, around 2.9 dBi. However, note that according to the IEEE 802.15.6 standard for WBAN services [5], these gains are still enough to use these devices as off-body antennas in the 2.42-GHz band for ISM wireless sensor applications. According to all these results, the reflection coefficient, radiation pattern and gain values, the prototype designed and optimized using the CRO-5SL algorithm presents the best performance at 2.42 GHz for ISM wireless sensor applications. Moreover, the textile material that has been used in the antenna manufacturing makes its embedding into clothes easy, with multiple real applications. Compared to other commercial solutions [43], our proposal presents a similar reflection coefficient and gain properties, but a reduced size.

### 4.1. A Note on the CRO-SL Algorithm Performance

The performance of the proposed CRO-SL in this optimization problem of textile MS-IFA design can be further analyzed by comparing its results with that of alternative state-of-the-art metaheuristics. Specifically, Table 4 shows the best results in terms of the objective function, given by Equation (1), obtained by the CRO-5SL (best CRO-SL version tested), with that of a harmony search approach, a differential evolution algorithm, a genetic algorithm with 2PX crossover and an evolutionary strategy with Gaussian mutation.

**Figure 13:**
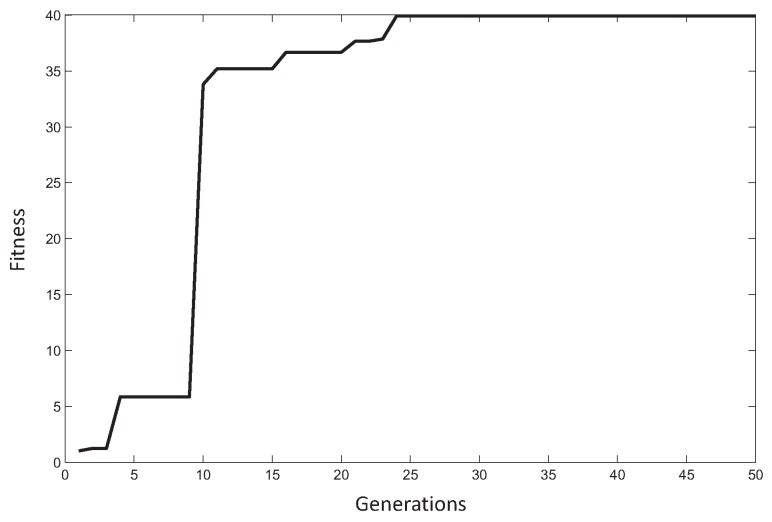
Best coral evolution in the CRO-5SL algorithm.

**Figure 14:**
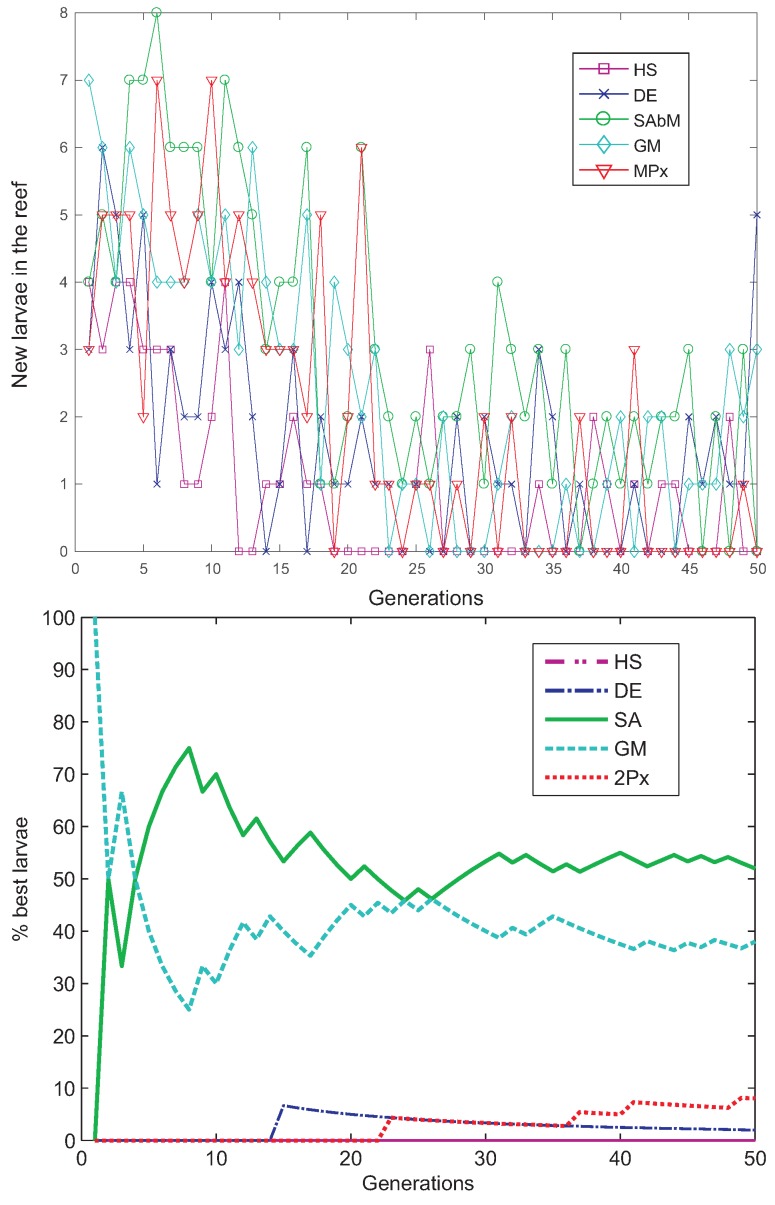
Evolution of the number of new larvae that are able to get into the reef per generation and substrate and the percentage of the best larvae obtained from each substrate, in the best run of the CRO-5SL algorithm: (**Top**) number of new larvae in the reef; (**Down**) percentage of best larvae formed.

Figure 13 shows the fitness evolution of the CRO-5SL when the best antenna prototype was obtained. As can be seen, the CRO-5SL approach is able to quickly obtain a high-quality solution, obtaining the algorithm’s convergence in less than 25 generations. Figure 14a,b illustrates an analysis of the CRO-5SL performance in terms of its different substrates. Figure 14a shows the evolution of the number of new larvae in the reef per generation and substrate. This figure can assist us in evaluating which substrates are able to get more corals (solutions) in the reef. As can be seen, the SA, GM and 2PX operators are the ones that attach a larger number of corals to the reef in each generation. It seems that the other operators are not able to attach so many larvae during the evolution. This is confirmed in Figure 14b, where the percentage of the best larvae formed during the evolution per substrate is displayed. As can be seen, the SA and GM substrates are able to generate the best larvae (solutions) consistently during the CRO-SL evolution. The 2PX and DE substrates obtain the best larvae in a small percentage of the generations, whereas it seems that the HS is not able to obtain the best larvae at any time. Note, however, that the HS is able to attach some solutions to the reef (as shown in Figure 14a and contributes in this way to CRO-SL’s good performance. The good performance of the SA operator in this problem is especially interesting. The SA operator, first introduced in [37], is able to introduce a kind of highly non-linear search pattern, with fractal structure, which seems to be very effective at obtaining good solutions in the optimization problem at hand.

## 5. Conclusions

A novel Coral Reefs Optimization with Substrate Layer (CRO-SL) algorithm has been proposed to adjust the resonant frequency and the bandwidth of the wearable antenna for off-body communications Wireless Body Area Network (WBAN) services at 2.42 GHz. The structure of the proposed device is based on an inverted-F antenna including a meander shape with variable width and spacing, manufactured with textile materials (felt and flexible copper). The CRO-SL algorithm has been used to establish the optimal antenna and meander dimensions, leading to an optimization of the characteristics of the resonant frequency and bandwidth of the MS-IFA. The algorithm has been implemented in MATLAB, and antenna simulations during the evolution have been carried out using the CST Microwave Studio software, which allows a complete simulation of tentative antenna prototypes and the fast calculation of the objective function. We have also shown the goodness of the proposed CRO-SL approach by comparing its performance to that of alternative state-of-the-art metaheuristics, obtaining better results in all cases. We have also shown that the proposed automatic optimization process proposed is able to obtain a high quality antenna for WBAN sensors in the ISM band, with good properties of bandwidth and resonant frequency, by constructing the best devices obtained from the CRO-SL evolution. The constructed antennas fully agreed with the simulations carried out in their design, and moreover, the final prototypes constructed present reduced dimensions and light weight, which are important characteristics of a wireless sensor ISM antenna to be embedded into protective clothing for different applications.

## Figures and Tables

**Table 1:** Variable values for the original IFA meander line antenna in [30].

Variable	Value (mm)	Variable	Value (mm)
$L_{s}$	52	$W_{s}$	44.5
$L_{f}$	31	$W_{f}$	1
$L_{L1}$	19	$W_{L1}$	1
$L_{L2}$	19	$W_{L2}$	2
$L_{L3}$	10.25	$W_{L3}$	1
$L_{L4}$	10.25	$W_{L4}$	1
$L_{L5}$	10.25	$W_{L5}$	1
$L_{L6}$	15	$W_{L6}$	1
$L_{S1}$	1	$W_{S1}$	5
$L_{S2}$	1	$W_{S2}$	4
$L_{S3}$	1	$W_{S3}$	6
$L_{S4}$	1	$W_{S4}$	4
$L_{S5}$	1	$W_{S5}$	6
$L_{S6}$	1	$W_{S6}$	4
$L_{S7}$	1	$W_{S7}$	6
$L_{S8}$	1	$W_{S8}$	4

**Table 2:** CRO-SL optimization parameters. GM, Gaussian Mutation; SA, Strange Attractors.

Phase	Parameter
Initialization	Reef size = 20×10
	$\rho_{0}=0.9$
External sexual reproduction	$F_{b}=0.80$
	T=5 substrates: HS, DE, 2PX, GM, SA
Internal sexual reproduction	1−Fb=0.20
Larvae setting	κ=3
Asexual reproduction	$F_{a}=0.05$
Depredation	$F_{d}=0.15$
	$P_{d}=0.05$
Stop criterion	$k_{max}=50$ iterations.

**Table 3:** Antenna dimensions after the optimization process with CRO-4SL and CRO-5SL.

Variable (mm)	Original	CRO-4SL	CRO-5SL
$L_{s}$	52	52	52
$W_{s}$	44.5	44.5	44.5
$L_{f}$	31	31.01	29.76
$W_{f}$	1	1	1
$L_{L1}$	19	18.93	19.67
$W_{L1}$	1	1	1.10
$L_{L2}$	19	19.74	20.66
$W_{L2}$	2	2.97	3.47
$L_{L3}$	10.25	17.5	13.06
$W_{L3}$	1	1	5
$L_{L4}$	10.25	17.5	13.06
$W_{L4}$	1	1	4
$L_{L5}$	10.25	17.5	13.06
$W_{L5}$	1	1	4
$L_{L6}$	15	0	5.96
$W_{L6}$	1	1	1.73
$L_{S1}$	1	0.81	0.87
$W_{S1}$	5	5	5
$L_{S2}$	1	1.19	1.13
$W_{S2}$	4	5	5
$L_{S3}$	1	17.5	0
$W_{S3}$	6	6	0
$L_{S4}$	1	1.19	1
$W_{S4}$	4	6	6
$L_{S5}$	1	17.5	1.13
$W_{S5}$	6	6	6
$L_{S6}$	1	1.19	1.13
$W_{S6}$	4	6	6
$L_{S7}$	1	17.5	0
$W_{S7}$	6	6	0
$L_{S8}$	1	0.99	1.13
$W_{S8}$	4	5	0

**Table 4:** Comparative results of the CRO-5SL and alternative metaheuristic performance (in terms of the objective function given by Equation (1)).

Algorithm	Best Fitness
CRO-5SL	39.9009
HS	9.4808
GA (2PX crossover)	9.4376
ES (GM)	11.0827
DE	9.8170
